# New therapeutic target for pediatric anaplastic ependymoma control: study of anti-tumor activity by a Kunitz-type molecule, Amblyomin-X

**DOI:** 10.1038/s41598-019-45799-4

**Published:** 2019-07-10

**Authors:** Lorena Favaro Pavon, David Capper, Tatiana Tais Sibov, Silvia Regina Caminada de Toledo, Ulrich-W. Thomale, Jean Gabriel de Souza, Francisco Romero Cabral, Carolina Maria Berra, Marcos Devanir Silva da Costa, Jardel Mendonça Niçacio, Patrícia Alessandra Dastoli, Daniela Mara de Oliveira, Suzana M. F. Malheiros, Edgar Ferreira da Cruz, Jackeline Moraes Malheiros, Sérgio Mascarenhas de Oliveira, Nasjla Saba Silva, Antonio Sérgio Petrilli, Andrea Maria Cappellano, Milena Colò Brunialti, Reinaldo Salomão, Manoel A. de Paiva Neto, Ana Marisa Chudzinski-Tavassi, Sérgio Cavalheiro

**Affiliations:** 1 Discipline of Neurosurgery, Federal University of São Paulo, São Paulo, São Paulo Brazil; 2 Charité -Universitätsmedizin Berlin, corporate member of Freie Universität Berlin, Humboldt-Universitätzu Berlin, and Berlin Institute of Health, Department of Neuropathology, Berlin, Germany; 3 German Cancer Consortium (DKTK), Partner Site Berlin, German Cancer Research Center (DKFZ), Heidelberg, Germany; 4 Pediatric Oncology Institute, Grupo de Apoio ao Adolescente e à Criança com Câncer (GRAACC), Federal University of São Paulo, São Paulo, São Paulo Brazil; 5 Pediatric Neurosurgery, Campus Virchow Klinikum, Charité Universitätsmedizin, Berlin, Germany; 6 Laboratory of Molecular Biology, Butantan Institute, São Paulo, São Paulo Brazil; 7 Centre of Excellence in New Target Discovery (CENTD), Butantan Institute, São Paulo, São Paulo Brazil; 8 Hospital Israelita Albert Einstein, São Paulo, São Paulo Brazil; 9 Department of Pharmacology, Institute of Biomedical Science, University of São Paulo, São Paulo, São Paulo Brazil; 10 Department of Genetics and Morphology, University of Brasília, Brasilia, Brazil; 11 Discipline of Nephrology, Federal University of São Paulo, São Paulo, São Paulo Brazil; 12 Carlos Institute of Physics, São Paulo University, São Carlos, São Paulo Brazil; 13 Laboratory of Immunology and Infectology, Federal University of São Paulo, São Paulo, São Paulo Brazil

**Keywords:** CNS cancer, Biologics, Paediatric cancer

## Abstract

EPNs comprise a heterogeneous group of neuroepithelial tumors, accounting for about 10% of all intracranial tumors in children and up to 30% of brain tumors in those younger than 3 years. Actually, the pattern therapy for low-grade EPNs includes complete surgical resection followed by radiation therapy. Total surgical excision is often not possible due to tumor location. The aim of this study was to evaluate, for the first time, the anti-tumor activity of Amblyomin-X in 4 primary cultures derived from pediatric anaplastic posterior fossa EPN, Group A (anaplastic, WHO grade III) and one primary culture of a high grade neuroepithelial tumor with MN1 alteration, which was initially misdiagnosed as EPN: i) by *in vitro* assays: comparisons of temozolomide and cisplatin; ii) by intracranial xenograft model. Amblyomin-X was able to induce cell death in EPN cells in a more significant percentage compared to cisplatin. The cytotoxic effects of Amblyomin-X were not detected on hFSCs used as control, as opposed to cisplatin-treatment, which promoted a substantial effect in the hAFSCs viability. TEM analysis showed ultrastructural alterations related to the process of cell death: mitochondrial degeneration, autophagosomes and aggregate-like structures. MRI and histopathological analyzes demonstrated significant tumor mass regression. Our results suggest that Amblyomin-X has a selective effect on tumor cells by inducing apoptotic cell death and may be a therapeutic option for Group AEPNs.

## Introduction

Ependymoma (EPN) is a central nervous system (CNS) malignant illness of the ventricular system walls, being the third most common pediatric brain tumor^1^. The most common location, in children, is in the posterior fossa, after that the cerebral hemispheres. Over the past decade, posterior fossa EPN has been much investigated as the common cause of injury and death in children^2,3^.

EPNs has been the cause of 5% of all brain and CNS tumors in children and teenagers compared with 2% of adult patients^3^. EPNs are traditionally classified by the World Health Organization (WHO) as grades I, II and III according to the anaplasia grade. Clinical and research observations have been challenging the concept that prognosis can be done solely based on classical histological grades^4^. Age groups, EPN location, and molecular genetic alteration have a strong impact on the prognosis^5,6^. Pajtler *et al*.^6^ set a EPNs constant molecular classification using DNA methylation profiling. Nine subgroups were acknowledged in a cohort of 500 tumors, three in each CNS structural section, supratentorial (ST), posterior fossa (PF) and spinal (SP). This new classification indicates genetic heterogeneity within the same histological grade in which determines EPN behavior, therefore the tumor prognosis.

Pavon *et al*.^7^ set anaplastic EPNs primary cell culture (WHO grade III) contained in the posterior fossa (PF), from 1–10-year-old patients EPNs. Immunophenotypic pluripotency markers profile (CD133, CD90, SSEA-3 and CXCR4) was characterized and set an EPN experimental model, through intracerebroventricular infusion of EPN cells, which was able to replicate the histopathological characteristics of the original tumor with preservation of phenotypic features, of patient tumor, containing pseudorosettes, a histological hallmark of EPN.

Currently, the standard therapy for EPN includes radical surgical removal followed by radiation therapy. Due to the tumor location and the damage surrounding brain, it is common the non-possibility of radical surgical resection. EPN presents a remarkable resistance to nonsurgical conventional therapies (chemo and radiotherapy) and its treatment remains challenging. To date, there has been no effective treatment for ependymoma. Cisplatin has a modest response rate of 20–30%^8,9^. Meco *et al*.^10^ described EPN stem-like cells sensitive to temozolomide treatment by its inherent cell differentiation-inducing capability; however, the differentiated EPN stem cells acquired resistance to this agent. Therefore, new therapeutic options are needed.

Amblyomin-X, a homolog of Kunitz-type protein recognized in the salivary glands transcriptome from adult *Amblyomma cajennense* tick, presented anticoagulant and antiangiogenic properties, and also antitumor activity via induction of apoptosis and reticulum stress^11–14^. Current studies have demonstrated the Amblyomin-X cytotoxicity in different human tumor cells lines such as pancreatic (Panc1, AsPC1, BxPC3) and melanoma (SK-MEL-5 and SK-MEL-28)^13,15^. Particularly, cytotoxicity was not detected on non-tumor cells treated with Amblyomin-X, suggestive of discrimination for cancer cells.

This molecule’s Kunitz-type domain is similar to that of the endogenous tissue factor pathway inhibitor (TFPI)^12^ has anticoagulant, antigionenic and antitumor properties^16,17^. Amblyomin-X also acts by non-hemostatic mechanisms, such as proteasome inhibition^10,14^.

The aim of this study was to evaluate the antitumor activity of Amblyomin-X in pediatric anaplastic EPN primary culture and intracranial xenograft model, using the following strategy: i) the establishing of EPN primary culture up to the fourth cell passage; (ii) evaluation of the cellular cytotoxic effect of Amblyomin-X on human amniotic fluid stem cells (hAFSCs) and EPN primary culture by MTT assay for cell viability, flow cytometry using FITC Annexin V Apoptosis Detection Kit with 7-AAD; (iii) EPN primary culture labeled with nanoparticles; iv) experimental model by stereotactic intracerebroventricular infusion of labeled EPNs cells from 1–10 year-old patients; v) evaluation of the cytotoxic effect of Amblyomin-X in animal model by magnetic resonance imaging (MRI) and histological analysis of tumor tissues.

## Results

EPN cells (n = 5) were previously characterized by pathological and molecular assay and one case was rediagnosed as high-grade neuroepithelial tumor with MN1 alteration; in the sequence, research was continued with the investigation by following approach:

### Amblyomin-X induces cytotoxicity in EPN cells

**Figure 1:**
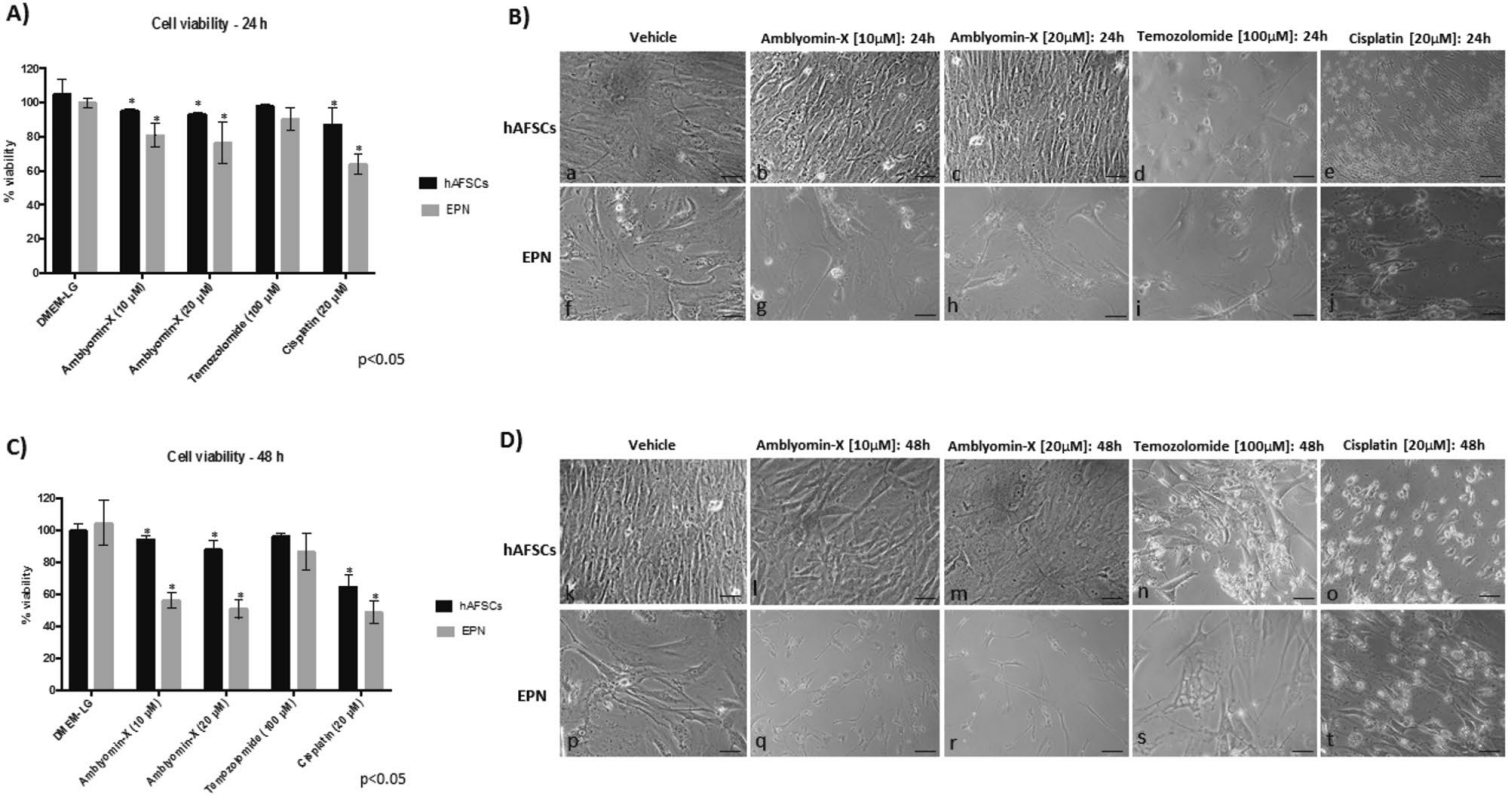
(**A**) Cell viability assay of hFSCs and EPN cells treated with DMEM-LG (vehicle), Amblyomin-X (10 and 20 µM), Temozolomide (100 µM) and Cisplatin (20 µM) for 24 h. (**B**) Cytological aspects of hFSCs (b-e) and EPN cells (g-j) treated with Amblyomin-X (10 and 20 µM), Temozolomide (100 µM) and Cisplatin (20 µM) for 24 h of treatment. (**C**) Cell viability assay of hFSCs and EPN cells treated with DMEM-LG (control), Amblyomin-X (10 and 20 µM), Temozolomide (100 µM) and Cisplatin (20 µM) for 48 h. (**D**) Cytological aspects of hFSCs (l-o) and EPN cells (q-t) treated with Amblyomin-X (10 and 20 µM), Temozolomide (100 µM) and Cisplatin (20 µM) for 48 h of treatment. Cytological aspects of hFSCs (B.a;D.k) and EPN cells (B.f;D.p) no treated. Scale: 100 µm.

Our first stage was to estimate Amblyomin-X effects *in vitro*, by comparing its viability’s effect in the EPNs primary cell culture and hAFSCs culture as normal cells control. Both EPNs and hAFSCs cells had a time-dependent and Amblyomin-X concentration-dependent (Fig. 1B,D).

Morphology study of EPN cells treated 24 h with Amblyomin-X shown minor changes and cell adhesion damage (Fig. 1B). Cells treated in forty-eight hours were certified as cell contraction, adhesion damage, inter-cell elongations loss and dispersed cell aggregates formation (Fig. 1D.q,r). EPN control cell (treated with vehicle in the same experimental conditions), exhibited a homogeneous culture distribution on the plate, exhibited a fusiform arrangement and multidirectional bundles set (Fig. 1B.f;1D.p). Conversely, when hAFSCs were evaluated under the same conditions, was not observed cytotoxic effect (Fig. 1A.a–e;1D.k–o). Furthermore, cells morphology did not change even after 24 h (Figures b,c) and 48 h of treatment (Fig. 1D.l,m), i.e., these cells remained with their original morphological fibroblastic-like characteristics (Fig. 1B.a;1D.k). Analysis of cell morphology after 24 and 48 h of treatment using temozolomide or cisplatin revealed morphological changes in both cell types (Fig. 1B.d,e,i,j;1D.n,o,s,t). Significant cytotoxic effects were observed on hAFSCs (Fig. 1B.d,e;1D.n,o), specially inter-cell elongations loss and dispersed cell aggregates formation using cisplatin (Fig. 1B.e;1D.o).

These results corroborated by cells viability assays, which demonstrate that after 24 h (Fig. 1A) and 48 h (Fig. 1C) of treatment with Amblyomin-X (10 µM and 20 µM) the EPNs cells display approximately 80% and 55% of viability, respectively. Cytotoxic effects of Amblyomin-X observed in EPN cells were more significant than observed after incubation with temozolomide for 48 h, 90% of viability.

Amblyomin-X cytotoxic effects were reproduced in five samples used in study: four EPN cells lines and CNS high grade neuroepithelial tumor with MN1 alteration, in which we choose to keep in the study considering the important effect of Amblyomin-X on high grade tumor cells by induce apoptotic cell death. It is worth mentioning that similar results were observed when we used cells derived from a neuroepithelial tumor that can be easily misdiagnosed when the molecular and epigenetic characterization is not performed.

### Cell apoptosis analysis by Flow Cytometry

Induction of apoptosis after treatment with Amblyomin-X (10 and 20 µM) and cisplatin (20 µM) was evaluated in EPNs and hFSCc for 24 and 48 h by FITC Annexin V/7AAD.

**Figure 2:**
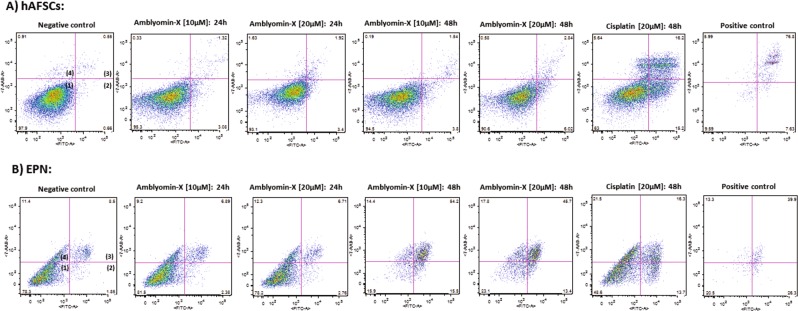
Induction of apoptosis by Amblyomin-X in (**A**) hFSCs and (**A**) EPN cells. The cells were incubated with 10 and 20 µM Amblyomin-X in DMEM-LG for 24 and 48 h, Cisplatin (20 µM) for 48 h and analyzed by flow cytometry using 7AAD and FITC-conjugated Annexin-V. It is worth mentioning that similar results were observed when we used cells derived from a neuroepithelial tumor. Quadrants 1–4: viable cells; early apoptotic cells; late apoptotic or necrotic cells; dead cells, respectively. Negative control: treatment with DMEM-LG; positive control to apoptosis: treatment with DMEM-LG and H_2_O_2_ [30%**]**.

The percentages of cell death in EPN observed after 24 h and 48 h of treatment with 20 µM Amblyomin-X were: early apoptotic cells (2,76%; 13,4%), late apoptotic or necrotic cells (6,71%; 45,7%), dead cells (12,3%; 17,8%), respectively (Fig. 2B). These results were more substantial than the results observed after treatment with cisplatin (20 µM) for 48 h: 13,7% early apoptosis, 16,3% late apoptosis or necrosis and 21,5%, death (Fig. 2B).

Our results show that Amblyomin-X induced cell death in EPN cells and, more significantly, were not observed cytotoxic effects in hFSCs (Fig. 2A), in which were used as normal control.

However, the treatment with cisplatin (20 µM) affected hFSCs viability considerably. After 24 h and 48 h of treatment with cisplatin (20 µM) the cells display approximately 90% and 70% of viability, respectively (Fig. 1A,C); and the percentages of cell death obtained after 48 h of treatment were the follow: 15,2% early apoptosis, 16,2% late apoptosis or necrosis and 5,64% death (Fig. 2A).

### EPN cells ultrastructural characterization

Ultrastructural characterization was performed in EPNs cell treated with Amblyomin-X (1.0 µM) for 48 h and untreated EPNs cell (vehicle control).

**Figure 3:**
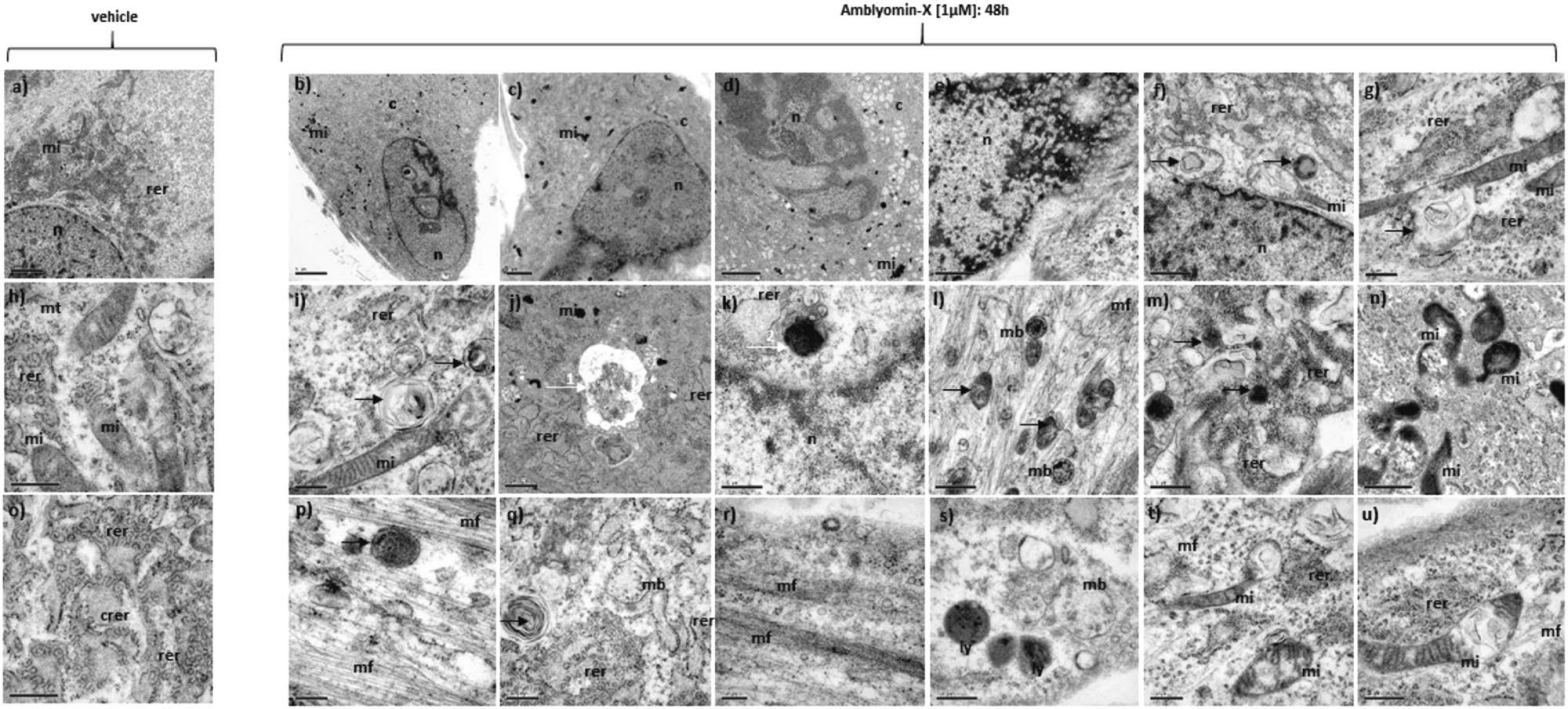
TEM of a primary cell culture of human EPNs treated with DMEM-LG (vehicle) (a, h, o) and primary cell culture of human EPNs treated (b-g, i-n, p-u) with Amblyomin-X (1.0 µM) in DMEM-LG for 48 h. n = nucleus; c = cytoplasm, nu = nucleoli; mi = mitochondria; rer = rough endoplasmic reticulum; crer = cisternae of rough endoplasmic reticulum; mt = microtubules; mf = microfilaments; mb = multivesicular bodies; ly = secondary lysosomes; black arrow = autophagosomes; white arrow 1 = aggregate-like structures; white arrow 2 = putative aggresomes. Scale: (a) 5.0μm; (b, c, d) 2.0μm; (e, f) 1.0μm; (g-u) 0,5 µm.

Untreated EPN cells analysis presented the following cytological description, spindle morphology and oval nuclei (Fig. 3a). The cell nucleus contains heterochromatic bundles and nucleoli (Fig. 3a). EPN cells exhibited significantly the presence of mitochondria with evident crests (Fig. 3h) and rough endoplasmic reticulum organized in tube-like structures known as cisternae (Fig. 3o).

Analysis of the EPN cells after Amblyomin-X treatment demonstrate various ultrastructural alterations compared to the control: *i*) altered pattern of nuclear heterochromatin condensation, initial appearance of pyknotic-like (Fig. 3b–d; *ii*) significant presence of mitochondria without evidence of crests, from small electron-dense (Fig. 3b–d,n) to large elongated (Fig. 3f-I,t,u) which described ultrastructural degeneration (Fig. 3f-i,t,u); *iii*) rough endoplasmic reticulum with disarranged cisternae (Fig. 3g,I,m,q,t,u); *iv*) presence of large number of autophagosomes or autophagy corpuscle (Fig. 3f-i,l,m,p,q); *v*) multivesicular bodies or late endosomes with eletron-denses (Fig. 3l) or no eletron-denses vesicles (Fig. 3q,s); *vi)* numerous microfilament (Fig. 3l,p,r,t,u); *vii)* presence of secondary lysosomes (Fig. 3s); *viii)* vesicular transport systems (Fig. 3r,u); *ix)* aggregate-like structures (Fig. 3j); *x)* putative aggresomes (Fig. 3k).

### EPN-mass was reduced by Amblyomin-X treatment

**Figure 4:**
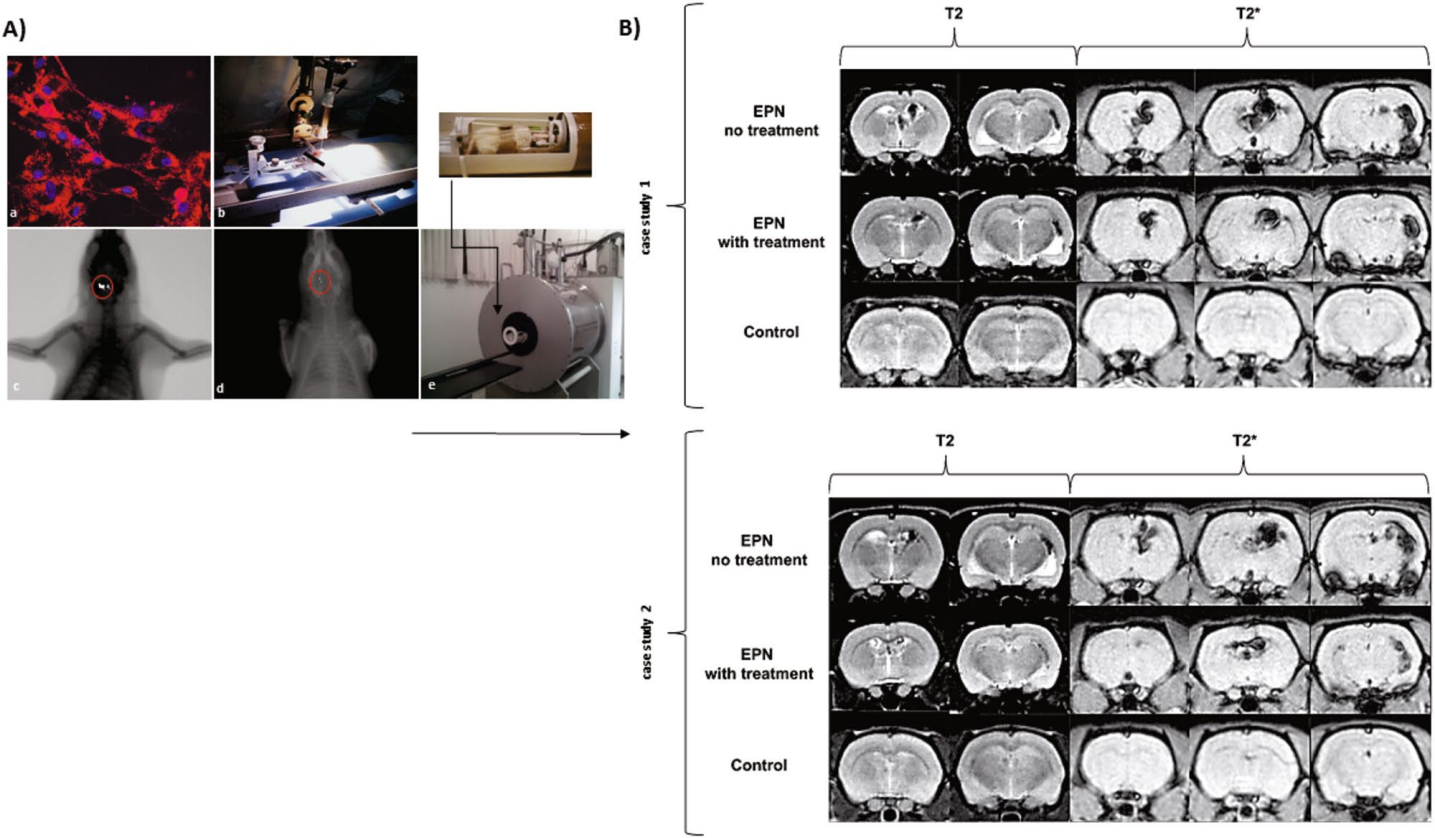
(**A**) a) Detection of MION-Rh labeled EPN cells by fluorescence assay. Magnification: 100×. (**A**) b) Stereotaxic implantation of MION-Rh labeled EPN cells in brain tumor experimental models. (**A**) c) Combined fluorescence and X-Ray tomography for *in vivo* detection of tumor (vehicle - saline) generate by infusion of MION-Rh labeled EPN cell. (**A**) d) Combined fluorescence and X-Ray tomography for *in vivo*detection of tumor treated with Amblyomin-X. (**A**) e) Radiofrequency bobine of animal positioning of the MRI neuroimaging equipment (2 Tesla): superconducting magnet 85310HR. (**B**) MRI monitoring of *in vivo* EPNs with no treatment (vehicle) and EPN with treatment (Amblyomin-X) of the case study 1 and 2 of the animals. Controls used are without any EPN cell transplantation, only sterile saline solution. Cases studies 1 and 2 represent fifteen independent experiments were performed. It is worth mentioning that similar results were observed when we used cells derived from a neuroepithelial tumor.

EPN cells were labeled with multimodal iron oxide nanoparticles conjugated with Rhodamine-B (MION-Rh), in order to be visualized in tumorigenicity tests, MRI and fluorescence imaging. MION-Rh nanoparticles were internalized by EPN cells and form intracellularly granules or fluorescent red clusters (Fig. 4A.a).

After EPN cells (MION-Rh) stereotaxic implantation (Fig. 4A.b) into the third ventricle, we observe tumor growth progression, in which it was viewed by fluorescence and X-Ray analysis. After 45 days, the tumor was detected due to fluorescence of the labeled cells (Fig. 4A.c). After 21 days of treatment with Amblyomin-X (1.0 mg/kg/day) by intra-peritoneal injection, we observed a significant decrease in the fluorescence signal indicating a tumor mass regression (Fig. 4A.d). Tumor growth was examined using MRI test (Fig. 4A.e;4B).

T_2_-weighted images exhibited hypointense zones in third ventricle, with subsequent ventricular dilatation, in which were more evident in the T_2_^*^-weighted images in the same area. Control group showed no sign of alteration during MRI.

These results detected significant tumor mass regression (Fig. 4A.c,d;4B) by comparing the two groups: EPN not treated, with EPN treated with Amblyomin-X (1.0 mg/kg/day).

### Histological analysis of EPN’s-brain after *in vivo* Amblyomin-X treatment

**Figure 5:**
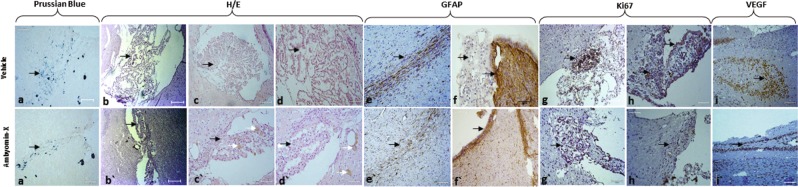
Immunopathological tumor analysis. a-i) EPN treated with vehicle. a’- I’) EPN with treatment of Amblyomin-X. a, a’) Histochemical analysis with Prussian Blue for MION-Rh detection in EPN cells.b-d; b’-d’) Hematoxylin and eosin staining.e,f;e’, f’) Immunohistochemical assay of tumor for glial fibrillary acidic protein (GFAP). g,h; g’-h’) Immunohistochemical assay of tumor for Ki67 for proliferation cellular detection. i, i’) Immunohistochemical assay of tumor for VEGF. White arrow: hemosiderin granules. Scale: 50 μm.

Histological, histochemical for Prussian Blue (EPN cells labeled with MION-Rh) and immunohistochemical staining, of rat brains (Fig. 5), demonstrated that EPN that has receive only vehicle exhibited high cellularity located in the third ventricle (Fig. 5b–d), invading periventricular areas (Fig. 5a,e–h) and proliferating through surrounding regions (Fig. 5g,h). Immunohistochemical analysis for the expression of human GFAP confirmed the glial origin of tumor (Fig. 5f), Ki67 detection shown high number of cycling cells in tumor tissue (Fig. 5g,h) and high expression of vascular endothelial growth factor (VEGF) in tumor.

Analysis of rats that received Amblyomin-X (1.0 mg/kg/day by intra-peritoneal injection) treatment (Fig. 5 a’-I’) revealed tumor mass regression, when compared control group (vehicle) (Fig. 5a–i), for example: decreased tumor mass in the ventricular space (Fig. 5b’-d’, f‘–h’) and in the brain parenchyma (Fig. 5e,f’).

## Discussion

EPNs are one of the most severe neoplasms in Pediatric Oncology because it presents an important pluripotentiality profile, which justifies the lack of efficient chemotherapy agents in the treatment. The long-term clinical outcome of pediatric intracranial EPN is poor with a high rate of recurrence. Therefore, effective therapeutic agents are urgently needed.

We evaluated, for the first time, the potential antitumor therapeutic application of Amblyomin-X in EPN primary culture.

Amblyomin-X is a recombinant protein identified on cDNA library from salivary glands of *Amblyomma cajennense* tick, which have shown antitumor, antiangiogenic and anticoagulant properties^18^.

Herein, Amblyomin-X treatment was able to induce cell death on EPN cells, but, excitingly, it did not decrease the viability of normal cells, preserving their original morphological characteristics of fibroblast-like cell type. On the other hand, the morphological and viability analysis of hAFSCs under temozolomide or cisplatin treatment revealed significant changes induced by cytotoxic effects, such as, dispersed cell aggregates formation and inter-cell elongations loss.

Concerning selective potential of Amblyomin-X by tumor cells, our results corroborated previous data, which demonstrate that Amblyomin-X acts on tumor cells with low toxicity in normal cells^17^. In addition, this great advance was confirmed for EPN’s, where the compared treatment with other current drugs preconized its tumor was superior^19–22^. For example, Phi *et al*.,^23^ have been used, as cells control, neural stem cells (NSCs) derived from the neocortex and immortalized astrocytes of the treatment with STAT3 inhibitors, which exhibit low viability compared to our results, i.e. 90–95% of hAFSCs viability.

Rudà *et al*.,^22^ described temozolomide as recover treatment for recurrent intracranial EPNs; however, complete response was achieved in only 5% of the patients and with median progression-free survival of 9.69 months. Platinum-based regimens is another treatment option, yet limited, in EPNs^21^.

In our studies, morphological analysis of EPNs cells after 24 h of Amblyomin-X treatment displayed minor changes or cell adhesion damage. Cells treated in forty-eight hours were certified as cell adhesion damage, dispersed cell aggregates formation and inter-cell elongations loss, compared to EPN control cell (vehicle). Amblyomin-X was able to reduce EPN cell viability in a time and dose-dependent manner. These results indicates that Amblyomin-X is more effective against EPNs than temozolomide or cisplatin.

In addition, EPNs cells morphological aspects changes, the phosphatidylserine exposition and DNA degradation were evaluated by FITC Annexin V/7AAD assay, after treatment with Amblyomin-X. We found that Amblyomin-X was able to induce apoptosis selectively in the EPNs cells, in accordance with the findings studies in renal cell carcinoma (RCC)^24,25^.

MTT assay results and flow cytometry corroborate with ultrastructural investigation, which demonstrate various alterations involved on cell death process, i.e: pyknotic-like nucleus, degenerated aspect of numerous mitochondria, rough endoplasmic reticulum with disarranged cisternae, autophagosomes or autophagy corpuscle, late endosomes, aggregate-like structures and aggresomes. Another studies using Amblyomin-X treatment also revealed, by TEM, spherical-type aggresome formation and alteration in rough endoplasmic of the tumor cell lines^14,24^.

Preclinical evidence^25,26^ has shown that aggresomes and the autophagy pathway have a crucial compensatory role in the protein-clearance mechanisms that eradicate potentially toxic proteins to promote resistance to proteasome inhibitors and, hence, tumor survival.

Amblyomin-X was able to recognizing renal tumor mass in animal bearing and remained for 3 times more than healthy animal, which has excreted via renal system^25^. Additionally, in healthy animal were not observed cytotoxic effects when used the concentration 100 fold more in acute dose-Amblyomin-X treatment during preclinical assays^25^. Several previous data have evaluated the Amblyomin-X action on process of tumor cell death with modulations related to the cell cycle arrest and inhibition of the ubiquitin-proteasome system, disturbing its catalytic activity and leading to cell death via apoptosis^13–17,26^. Ours hypothesis could be that the same mechanism occurs in EPN cells. The present data concerning EPN’s-Amblyomin-X treated, have demonstrated series of intracellular events linked to cytotoxic effects leading to tumor cell death by induction aggresomes formation, as a possible non-exclusive ubiquitin pathway^27,28^. The power anticancer activity of Amblyomin-X on EPN cells compelled us to examinate this compound effectiveness *in vivo*, applying the established xenograft models^7^.

The intracranial xenograft model built from pediatric EPNs has showed an important approach to preclinical drug discovery^7^. Fluorescence, X-Ray and MRI detected tumor growth progression.

After 21 days of the daily treatment of EPN xenograft model with Amblyomin-X (1 mg/kg), by intra-peritoneal injection, we observed a significant tumor mass regression compared to the control (vehicle), in which was investigated by fluorescence, X-Ray, MRI and histological analysis.

MRI assay was extremely important to control tumor growth; T_2_-weighted images showed hypointense zones in third ventricle, with subsequent ventricular dilatation, in which were more evident in the T_2_^*^-weighted images in the same area. These results revealed tumor mass regression on the side of histopathological test, which confirmed reduction of dilatation of the third ventricle with decreased tumor cells proliferation in the same area.

These results are in accordance with the *in vitro* assays, were Amblyomin-X treatment have showed cytotoxic activity on EPN cells, induce cell death by apoptosis and a slight or no activity. Concluding, our results showed that Amblyomin-X acts selectively on EPN cells whereas it as a safety-potential anti-EPNs drug candidate.

Certainly, it would be of great importance the DNA methylation array study in molecular subtypes of EPN in both cell lines and tissue samples *in vivo*, building thus, a comparative profile among these subtypes and treatment with Amblyomin-X. Future trials will be developed, so we could lead to more precise prognostic assessments.

## Materials and Methods

Five human anaplastic EPNs (WHO grade III), located in the PF from 1–10 year-old patients, were submitted to resection at Pediatric Oncology Institute, Grupo de Apoio ao Adolescente e à Criança com Câncer (GRAACC), Department of Neurology and Neurosurgery, Federal University of São Paulo(UNIFESP), São Paulo, Brazil. All parent and/or legal guardians signed an informed consent for the study and all methods involving human samples were performed in accordance with the relevant guidelines and regulations (Ethical Committee in Research of Federal University of São Paulo_UNIFESP).

Recently, EPNs were classified according to the DNA methylation profile^6^, such classification showed to be increasingly dependent on molecular biology findings^29^. We highlight the WHO revised 2016 classification of CNS tumors^4^, which incorporates, for the first time, genetic information in addition to morphology for the classification of many tumor entities^30,31^.

As follows, both the pathologic anatomy and molecular diagnostics of EPNs were described in the Table 1. We performed molecular diagnostics through the methylation profile using Illumina DNA methylation arrays according Capper *et al*.^32^, in collaboration with *Institutfür Neuropathologie Charité - Universitätsmedizin Berlin, Germany*.

**Table 1:** Summary of immunohistochemical features and molecular diagnostics of the cell lines of patient tumors (n = 5).

Cell lines (n = 5)	Target phenotypes	Classifier Results
ALCMS (RH 80666)	KI 67 + (5%); GFAP + (50%); Nestin + (30%); p53 + (30%); MAP2 + ; EMA + ; VEGFR1 + (10%)	Ependymoma, PF – Group A (with low tumor cell content)
DSD (RH 80438)	Ki67 + (5%); GFAP + ; Nestin + (50%); p53 + (60%); MAP2 + ; EMA + ; VEGFR1 + (40%)	Ependymoma, PF –Group A
HBA (RH 87144)	Ki 67 + (20%); GFAP + ; Nestin + (40%); p53 + (70%); MAP2 + ; EMA + ; VEGFR1 + (30%)	Ependymoma, PF –Group A
VGM (RH 106159)	KI 67 + (30%); GFAP + (60%); Nestin + (30%); p53 + (60%); MAP2 + ; EMA + ; VEGFR1 + (40%)	Ependymoma, PF –Group A
YVSS (RH 90765)	Ki 67 (20%), papilliferous areas, EMA + in DOT, enolase, chromogranin A, INI-1 + , GFAP + , neurofilament -, P53 (50%), Ki 67 + (20%)	CNS high grade neuroepithelial tumor with MN1 alteration

### EPNs primary culture establishment

EPNs samples (n = 5) were washed in phosphate-buffered saline (PBS) (1×) followed by enzymatic dissociation with collagenase-I 0.3% (Sigma-Aldrich). The isolated cells were resuspended in Dulbecco’s Modified Eagle’s Medium-Low Glucose (DMEM-LG, Gibco/Invitrogen Corporation) and processed according Pavon *et al*.^7^.

### Collection Amniotic Fluid (AF) and culture of hAFSCs

We used hAFSCs as control samples, since this cell type is one of our ongoing scientific research projects. hAFSCs have been classified as a novel type of broadly multipotent/pluripotent stem cells sharing characteristics of both embryonic and adult stem cells^33^.

All pregnant women signed an informed consent for the study and all methods involving human samples were performed in accordance with the relevant guidelines and regulations (Ethical Committee in Research of Federal University of São Paulo_UNIFESP). Fetal AF (40 mL each) was obtained from six pregnant women with fetuses undergoing repair of Myelomeningocele (MMC), with gestational age of 26 weeks.

After collection the samples were kept in Dulbecco’s modified Eagle’s Medium-Low Glucose (DMEMLG; GIBCO/Invitrogen Corporation) supplemented with Antibiotic – Antimycotic 10,000 U/ml sodium penicillin, 10,000 µg/ml streptomycin sulfate, 25 µg/ml amphotericin B (GIBCO/Invitrogen Corporation) and processed within one hour. Fetal AF samples were diluted with DMEM-LG (1:1 v/v) (GIBCO Invitrogen/Corporation) and then centrifuged at 400 g and supernatants discarded. Cells pellet were resuspended in DMEM-LG (GIBCO/Invitrogen Corporation) supplemented with L-Glutamine 200 mM, Antibiotic- Antimycotic 10,000 U/ml sodium penicillin, 10,000 µg/ml streptomycin sulfate, 25 µg/ml amphotericin B (GIBCO/Invitrogen Corporation) and 10% Fetal Bovine Serum (FBS) (GIBCO/Invitrogen Corporation). The experiments described were performed with cells in the second or third cell passages. Immunophenotypic profile of hAFSCs were positive for the markers that characterize the profile of MSCs (mesenchymal stem cells), markers for pluripotency, homing and undifferentiation, according Sibov *et al*.^33^.

### Amblyomin-X

De Souza *et al*.^25^ described Amblyomin-X, a 15 kDa protein, obtained in a recombinant form.

### Cytotoxicity assays

#### Cell viability

The 3-(4,5-dimethylthiazol-2-yl)−2,5-diphenyltetrazolium bromide (MTT) assay was applied to assess the cell viability on primary cultures of EPNs (1 × 10^4^ cells/well) and hAFSCs (2 × 10^4^ cells/well) in the plate of 96 wells,according^12^. Briefly, the cells were treated with Amblyomin-X [10 or 20 µM]; Temozolomide [100 µM] (IC _50_ used to glioblastoma = 10 µM)^9^ or Cisplatin [20 µM]^19^, and incubated (37 °C, 5% CO_2_) for 24 or 48 hours. DEMEM-LG was used as negative control in equal volume and conditions of the other treatments.

### Cellular death by flow cytometry

The cellular death was evaluated in EPN and hAFSCs cells incubated with DMEM-LG (negative control), Amblyomin-X [10 µM or 20 µM], Cisplatin [20 µM] for 24 or 48 h. In addition, as positive control, the cells were incubated with H_2_O_2_ [30%] for 30 minutes. Amounts of 105 EPNs cells or hAFSCs were twice washed with cold PBS. To labeling procedure, the cells were resuspended in 100 µL of Binding Buffer 1× (Molecular Probes, Invitrogen, USA), and were added FITC Annexin V (Molecular Probes, Invitrogen, USA) and 7-Amino-Actinomycin D (AAD – BD Biosciences), 5 µL each. The samples were incubated during 15 minutes in the dark at room temperature. Posteriorly, the volumes were adjusted to 500 µL using Binding Buffer 1×. Data acquisition was performed within one hour of labeling in LSRFortessa flow cytometry (BD Biosciences, San Jose, CA, USA) and analyzed by the software FlowJo.

### Transmission Electron Microscopy (TEM)

#### EPNs primary culture establishment

was performed using ACLAR® film. In the sequence, the cells were treated with Amblyomin-X [1 µM] for 48 h. EPN cells adhered on ACLAR® film were fixed in 1% glutaraldehyde and processed according to Pavon *et al*.^7^. Semi and ultrathin sections were obtained with the aid of a Porter Blum ultramicrotome. The ultrathin sections (70 nm) were placed on copper grid, which were photographed under a TEM (Philips CM100).

### EPNs cells labeling with multimodal iron oxide nanoparticles (MION) conjugated to Rhodamine-B (Rh-B) (MION-Rh) *in vitro*

10^3^EPNs cells were plated in 24-well plates and incubated overnight, (for approximately 18 hours at 37 °C, 5% CO_2_) in DEMEM-LG medium, with 40 μg Fe/mL MION-Rh. After incubation, the culture medium solution was removed and the cells were washed twice with PBS (1×) to remove extracellular MION-Rh. The labeled cells were treated with 0.25% Tryple Express, and then harvested and manually counted using 0.4% Trypan Blue (Gibco/Invitrogen Corporation) and fixed with 4% paraformaldehyde. Next, fluorescence analysis were done using diamidino-2-phenylindole (DAPI, Sigma- Aldrich) to label the cell nuclei and an Rh-B filter (530 nm and 550 nm) to detect the MION-Rh, according Pavon *et al*.^7^. The Intracellular detection of MION-Rh in labeled EPNs cells were performed using a fluorescence microscope (IX51 Olympus, Tokyo, Japan).

### Animal ethics statement

Experimental procedures were performed in accordance with the guidelines for animal experimentation determined by the UNIFESP Care Committee. The Committee on the Ethics of Animal Experiments of the UNIFESP (CEUA n. 1098-09) approved this protocol. Ethical conditions were followed assuming all international rules of animal care outlined by the International Animal Welfare Recommendations and in accordance with local institutional animal welfare guidelines.

### Stereotaxic implantation of MION-Rh labeled EPN cells

The animals (n = 15; male Wistar rats) were treated with immunosuppressant drug (ciclosporin [10 mg/Kg/day]) during 45days. To implant of EPN cells, the animals were anesthetized with a solution containing ketamine (55 mg/kg) and xylazine (11 mg/kg) following, the hair in the top of the head was removed. The animal was fixed on stereotaxic apparatus (Stoelting^®^, model 51700). After skin incision on the dorsal region of skullcap, bone cap trepanation was made using a dental drill. According to Swanson’s Stereotaxic Atlas guidelines (1992), the implantation position was determined following coordinates: 0.8 mm anteroposterior, 1.4 mm laterolateral, and a depth of 3.8 mm, as described before^28^. Hamilton syringe was used to implant 10^4^ EPN cells in 10 µL of culture medium into the third ventricle. Tumor development was monitored over 45 days, according Pavon *et al*.^7^.

### *In vivo* image of tumor progression tracking

Tumor development were monitored using an *in vivo* imaging device, Bruker model MS FX PRO (Bruker, Ettlingen, GE). Throughout image acquisition, animals were placed in dorsal recumbence and remained anesthetized with inhaled 2% isoflurane in oxygen at 2 L/min. Initially, the skull images were acquired by X-ray. The fluorescence of the labeled cells was evaluated using the excitation (540 nm) and emission (585 nm) of MION-Rh. The images were acquired and evaluated using multiplex location software. The images were acquired and evaluated using multiplex-located software, according Pavon *et al*.^7^.

### Magnetic Resonance Imaging (MRI) tumor analysis

MRI brain scans were obtained in a 2 Tesla/30 cm horizontal superconducting magnet 85310HR (Oxford Instruments, Abingdon, UK) interfaced to a Bruker Avance AVIII console (Bruker-Biospin, Ettlingen, GE) with Paravision 5.1 software (Bruker). A Crossed Saddle radiofrequency coil was used as a head probe in animals anesthetized with ketamine/xylazine (95/12 mg/kg, i.p.). A T_2_-weighted RARE (Rapid Acquisition with Refocused Echoes) sequence (TR = 5000 ms, TE = 40.5 ms, RARE factor = 8, 4 averages, 6 minutes/animal) was used in a volume of 32 × 32 × 24 mm^3^ covered with a 128 × 128 matrix and 2 mm slice thickness without gaps (12 slices), generating a spatial resolution of 250 × 250 µm^2^. Immediately after RARE acquisition, a T2*- weighted image, using a FLASH (Fast Low Angle Shot) sequence (TR = 500 ms, TE = 15 ms, flip angle = 30°, 8 averages, 6 minutes/animal) was acquired^34^. For this image, a volume of 32 × 32 × 24 mm^3^ was covered with a 192 × 192 matrix and 2 mm slice thickness without gaps (12 slices), generating a spatial resolution of 167 × 167 µm^2^, according Pavon *et al*.^7^.

### Treatment of EPN xenograft model by Amblyomin-X

After tumor establishment, (3–5 mm diameter of the tumor mass) Amblyomin-X (1.0 mg/kg/day) treatment or vehicle (saline) by intra-peritoneal injection was performed during 21 days. The anti-tumor activity was assessed by *in vivo* image, MRI and histological analysis.

### Histological analysis of tumor tissues

After image acquisition, the animals were anesthetized and transcardially perfused with a buffered saline solution and 4% paraformaldehyde (PFA). The brains were removed and stored in PFA for 24 hours; they were then cryoprotected in a 40% sucrose solution for 48 hours. Subsequently, 40μm thickness coronal sections were cut using a cryostat (Leica) and stained using standard procedures for hematoxylin-eosin, Prussian Blue staining and for immunohistochemical staining was used human primary antibodies included the following: glial fibrillary acidic protein (GFAP) (1:200), Ki-67 (1:20) and VEGFR-1 (1:150) (Epitomics, Inc.), according Pavon *et al*.^7^.
